# Supplementary material for: Layer 5 myelination gates corticothalamic coincidence detection
Source: Nat Commun. 2025 Dec 11;16:10922. doi: 10.1038/s41467-025-66157-1 (PMC12699038; doi:10.1038/s41467-025-66157-1)
Supplement: Supplementary file 1 — Supplementary Information [file 41467_2025_66157_MOESM1_ESM.pdf]

Supplementary information

Layer 5 myelination gates corticothalamic coincidence detection

Nora Jamann, Jorrit S. Montijn, Naomi Petersen, Roeland Lokhorst, Daan van den Burg, Maayke Balemans, Stan L.W. Driessens, J. Alexander Heimel & Maarten H.P. Kole*

* Corresponding author; m.kole@nin.knaw.nl

Supplementary Figures 1 to 9

Supplementary Table 1

Supplementary Figures

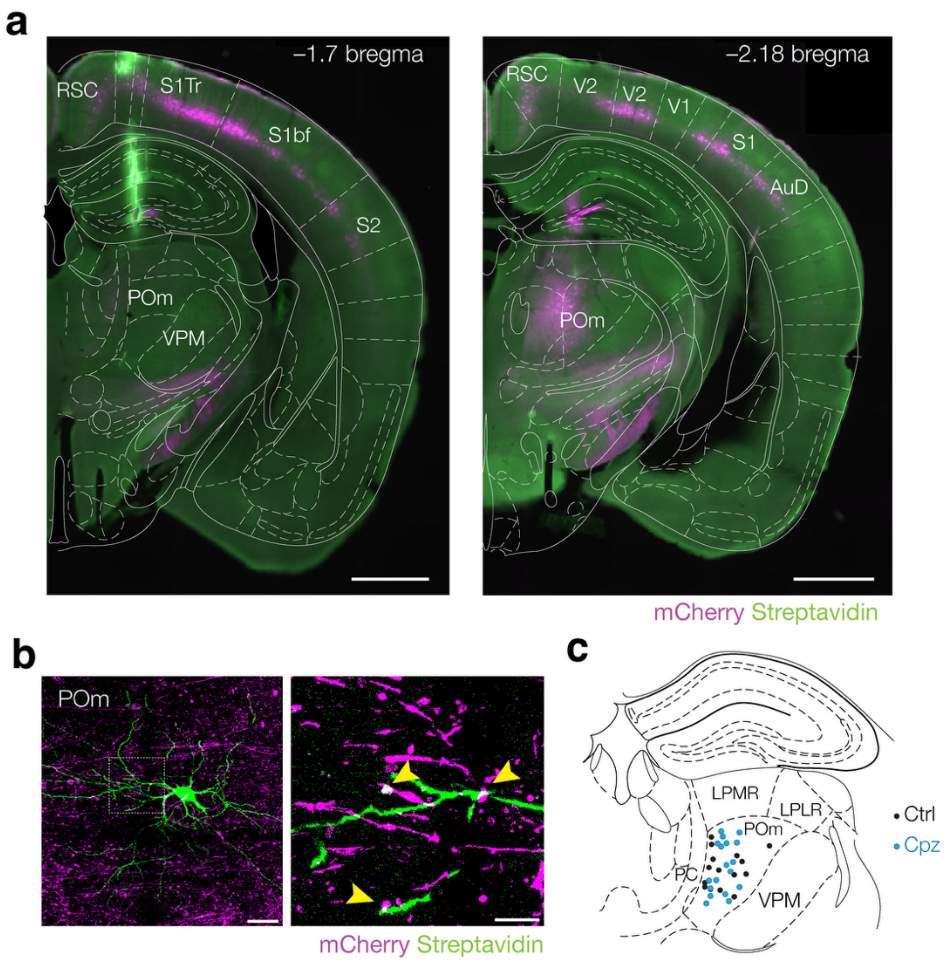

Supplementary Fig. 1 | Reconstruction of recording locations in the POm.

a Confocal image of coronal brain sections with pipette tracks from biocytin spill in the extracellular solution (Streptavadin, green) and mCherry^{+} fluorescence (magenta) overlaid with coronal brain atlas diagrams (adapted from Figures 47 and 49 in Ref. [104]). Scale bar, 1 mm.
b Confocal image of an opto-tagged juxtacellularly recorded POm neuron filled with biocytin (streptavadin, green) surrounded by mCherry^{+} L5 axons (magenta) and putative presynaptic terminals (yellow arrows). Scale bars, 30 µm (*left*), 10 µm (*right*).
c Overview of reconstructed locations of recovered neurons based on extrapolation of pipette tracks and noted *z*-depths of recording mapped onto a brain atlas diagram. VPM, ventral posteromedial thalamic nucleus; LPMR, lateral posterior thalamic nucleus, laterorostral part; LPLR, lateral posterior thalamic nucleus, mediorostral part; PC, paracentral thalamic nucleus. POm, posterior medial thalamic nucleus. Dots represent n = 14 recovered neurons, from N = 14 mice (Ctrl), n = 18 neurons, from N = 13 mice (Cpz).

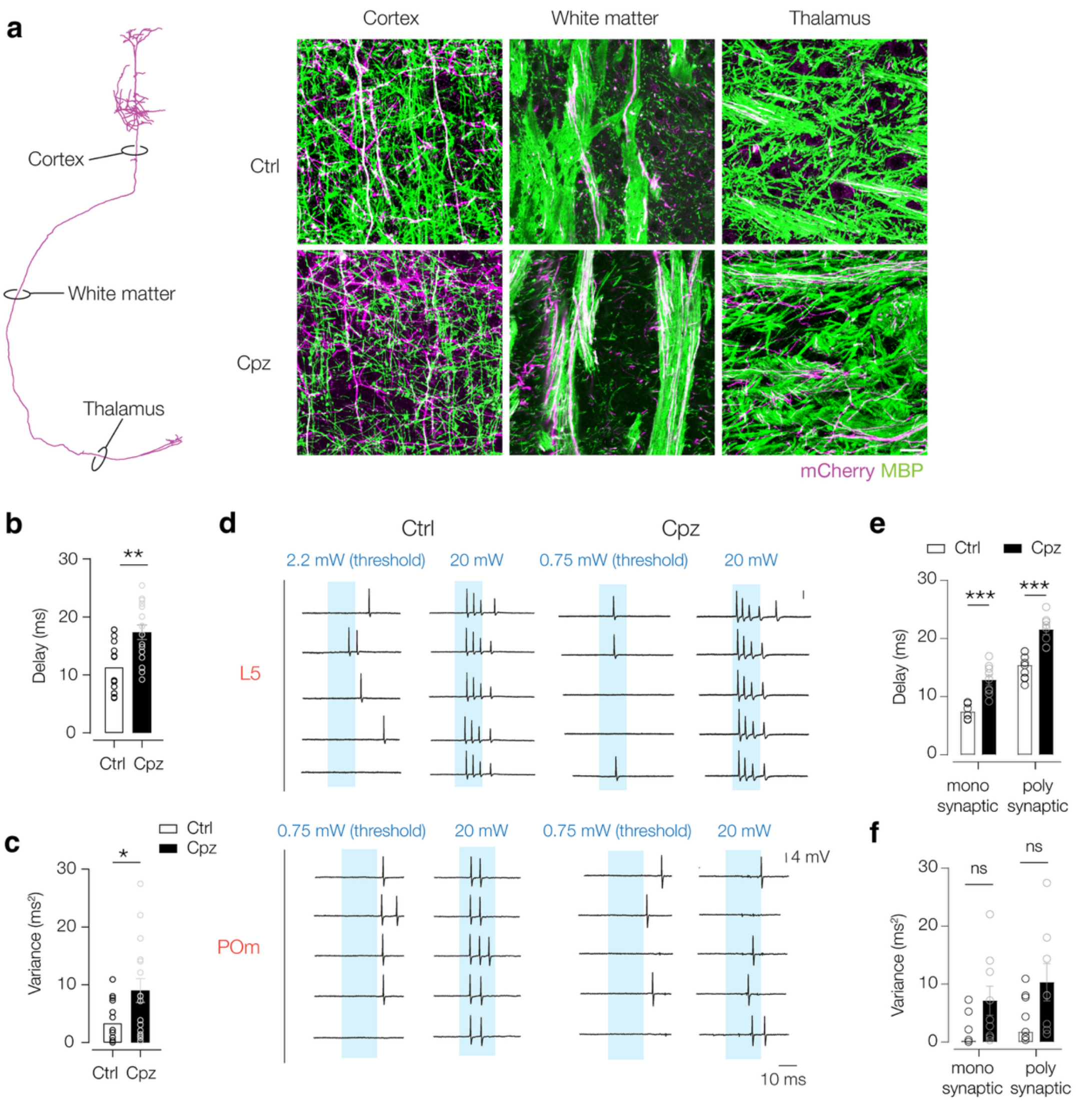

Supplementary Fig. 2 | Cortical demyelination delays L5–POm spiking independent of light output power or connection type.

a L5–POm example neuron and confocal images of myelination (MBP, green) in regions that mCherry+ axons (magenta) are traveling through in Ctrl and Cpz mice.

b Increased delay at 25 mW of Cpz (black) vs Ctrl (white) neurons. Unpaired t-test **P = 0.0013, t = 3.565, df = 29.

c Increased variance at 25 mW of Cpz (black) vs Ctrl (white) neurons. Unpaired t-test **P = 0.0022, t = 2.43, df = 29. For b, c; n = 15 neurons, N = 11 mice (Ctrl), n = 16 neurons, N = 10 mice (Cpz).

d 5 consecutive example trials of a Ctrl and Cpz neuron in L5 and POm respectively, recorded at low light power (threshold for spiking, left) and high light power (20 mW, right). Note the increased spike numbers, but lower delay and variance at high light power.

e Increased delay of spiking relative to light onset both in putative monosynaptic and polysynaptic connections in Cpz (black) vs Ctrl (white) neurons. 2-way ANOVA $P < 0.0001$, $F_{(1,30)} = 188.2$ connection type; $P < 0.0001$, $F_{(1,30)} = 62.10$ treatment; $P = 0.46$, $F_{(1,30)} = 0.561$ interaction. Šídák's multiple comparisons ***P < 0.001.

f Significant increase in variance of putative monosynaptic and polysynaptic connections for Cpz neurons (treatment effect) does not depend on connection type. 2-way ANOVA *P = 0.0127, $F_{(1,30)} = 7{,}026$ treatment; $P = 0.24$, $F_{(1,30)} = 1.445$ connection type; $P = 0.799$, $F_{(1,30)} = 0.066$ interaction. Šídák's multiple comparisons $P > 0.05$. For e, f**;** n = 17 neurons, N = 11 mice (Ctrl), n = 17 neurons, N = 10 mice (Cpz). Source data are provided as a Source Data file.

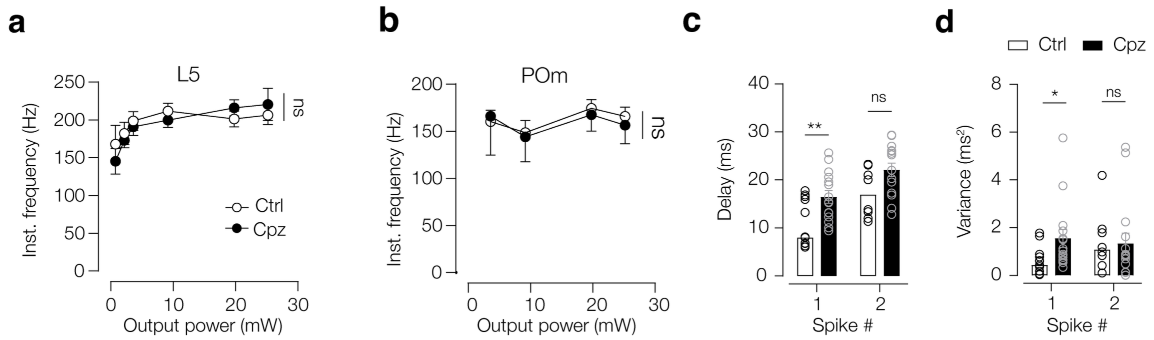

Supplementary Fig. 3 | Optogenetically evoked burst frequencies in L5 and POm are not affected by demyelination.

a Instantaneous frequency of light-evoked bursting in L5 pyramidal neurons was not changed in demyelination. 2-way ANOVA P = 0.66, $F_{(1,47)}$ = 0.199 treatment; P = 0.006, $F_{(5,47)}$ = 3.79 output power; P = 0.79, $F_{(5,47)}$ = 0.48 interaction. Šídák's multiple comparisons P > 0.05 for all comparisons. n = 8 neurons, N = 6 mice (Ctrl), n = 5 neurons, N = 4 mice (Cpz).
b Instantaneous frequency of bursting in POm neurons was not changed between treatment groups. 2-way ANOVA P = 0.799, $F_{(1,58)}$ = 0.065 treatment; P = 0.58, $F_{(3,25)}$ = 0.66 output power,; P = 0.99, $F_{(3,58)}$ = 0.037 interaction. Šídák's multiple comparisons P > 0.05 for all comparisons. n = 13 neurons, N = 11 mice (Ctrl), n = 12 neurons, N = 8 mice (Cpz).
c First spike in a burst in the POm was significantly delayed in Cpz neurons. 2-way ANOVA P = 0.0006, $F_{(1,47)}$ = 13.57 treatment; P = 0.0001, $F_{(1,47)}$ = 17.72 spike nr.; P = 0.71, $F_{(1,47)}$ = 0.14 interaction. Šídák's multiple comparisons **P = 0.0065.
d Variance is specifically increased for the first spike in a burst in Cpz neurons. Kruskal-Wallis test P = 0.0595, Dunn's multiple comparisons *P = 0.0483 Ctrl vs Cpz (spike nr. 1), P > 0.05 other comparisons. For c, d**;** n = 13 neurons, N = 9 mice (Ctrl), n = 15 neurons, N = 9 mice (Cpz). All data are provided as mean +/- SEM. Source data are provided as a Source Data file.

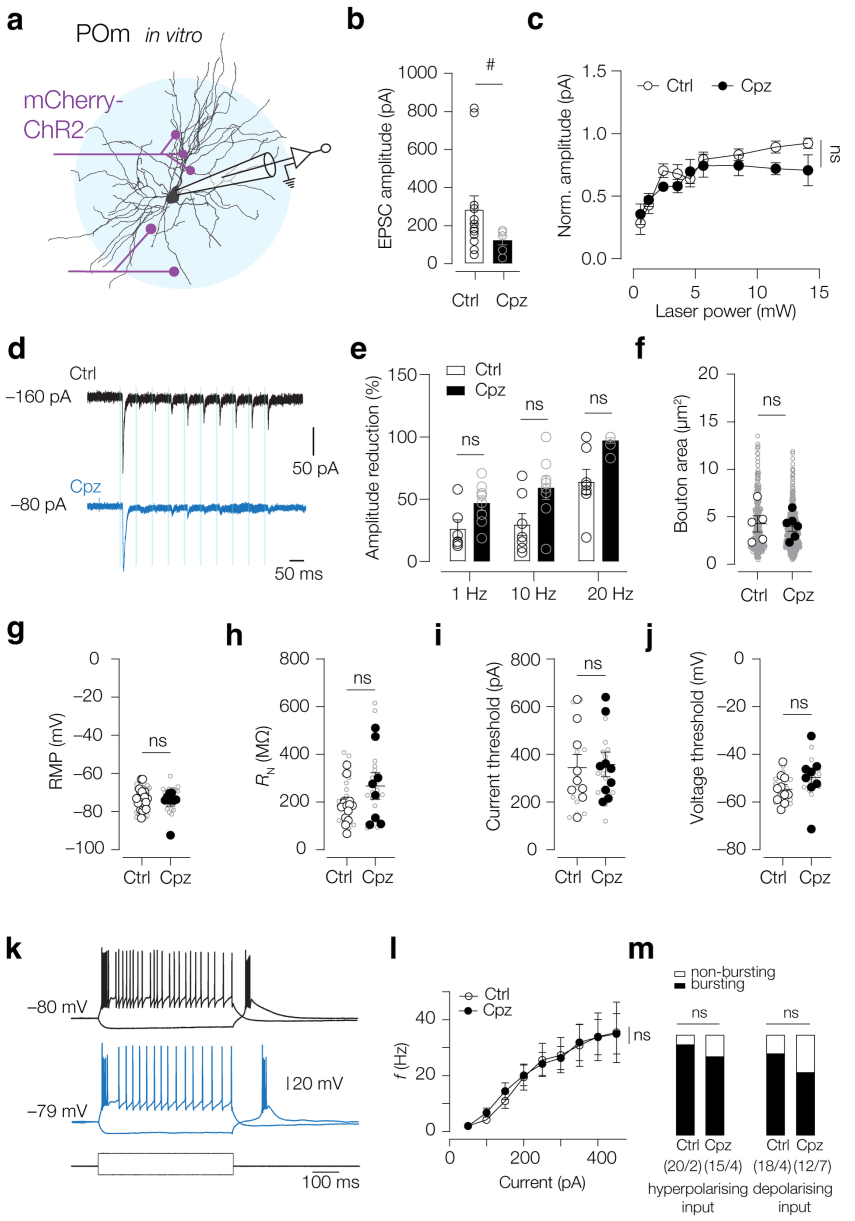

Supplementary Fig. 4 | *In vitro* evoked responses of L5–POm boutons as well as intrinsic firing properties are unchanged after demyelination.

a 3D reconstruction of a neuron recorded in whole-cell configuration *in vitro* and filled with biocytin for post-hoc localization. Neurons were stimulated with a laser (470 nm) above the soma and responses were recorded in voltage clamp.
b Maximum amplitude of evoked EPSCs during stimulation with a single stimulus (10 ms) was not significantly changed. Mann-Whitney test $^{\#}P = 0.0668$, n = 12 neurons, N = 7 mice (Ctrl), n = 6 neurons, N = 4 mice (Cpz).
c Normalized (to maximum response) amplitude in relation to the output light power was not different between groups. 2-way ANOVA $P < 0.0001$, $F_{(8,126)} = 11.32$ intensity; $P = 0.063$, $F_{(1,126)} = 3.511$ treatment; $P = 0.45$, $F_{(8,126)} = 0.99$ interaction. Šídák's multiple comparisons $P > 0.05$ for all comparisons. n = 11 neurons, N = 8 mice (Ctrl), n = 6 neurons, N = 4 mice (Cpz).
d Optical stimulation at 20 Hz (10 x 3 ms) evoked strong postsynaptic depression.
e Relative amplitude reduction of EPSCs at different stimulation frequencies, stimulation at 5 mW output power. No significant changes between Ctrl and Cpz neurons were observed (EPSC 10 versus EPSC 1). Kruskal-Wallis test $P = 0.0002$, Dunn's multiple comparisons $P > 0.05$ for all Cpz vs Ctrl comparisons. Facilitating cells were not included in the analysis.

f Bouton area of Ctrl and Cpz L5 axon boutons in POm was the same. Nested t-test P = 0.81, t = 0.25, df = 9, n = 291 boutons, N = 5 mice (Ctrl), n = 514 boutons, n = 6 mice (Cpz).
g Resting membrane potential (RMP) at *I* = 0 of Ctrl and Cpz POm neurons was not significantly different. Liquid junction potential (LJP) of –13 mV was corrected for. Mann-Whitney test P = 0.87. n = 41 neurons, N = 25 mice (Ctrl), n = 23 neurons, N = 9 mice (Cpz).
h Input resistance (R_N) of Ctrl and Cpz POm neurons was not significantly different. Nested t-test P = 0.13, t = 1.59, df = 21. n = 22 neurons, N = 15 mice (Ctrl), n = 20 neurons, N = 8 mice (Cpz).
i Current threshold for single AP generation of Ctrl and Cpz POm neurons was not significantly different. Nested t-test P = 0.96, t = 0.05, df = 16. n = 16 neurons (Ctrl), n = 20 neurons (Cpz), N = 9 mice (Ctrl & Cpz).
j Voltage threshold for single AP generation of Ctrl and Cpz POm neurons was not significantly different. LJP was corrected for. Nested t-test P = 0.22, t = 1.27, df = 16. n = 17 neurons, N = 10 mice (Ctrl), n = 16 neurons, N = 8 (Cpz).
k Current injection responses of a Ctrl (black) and Cpz (blue) neuron. Note the hyperpolarization-evoked rebound bursting as well as the low-current input depolarization evoked bursts. –100 pA and +250 pA current injections respectively.
l Input-frequency relationship was unchanged between Ctrl and Cpz neurons. Average frequency was determined over the entire 500 ms current step. 2-way ANOVA P = 0.83, $F_{(1,202)}$ = 0.046 treatment, P < 0.0001, $F_{(8, 202)}$ = 11.92 input current, P > 0.99 interaction, $F_{(8,202)}$ = 0.06. Šídák's multiple comparisons P > 0.99 for all comparisons. n = 15 neurons, N = 11 mice (Ctrl), n = 13 neurons, N = 7 mice (Cpz).
m *Left*: Fraction of hyperpolarization evoked burst-firing neurons. n indicated in figure. Fischer's exact test P = 0.39. *Right*: Fraction of depolarization evoked burst-firing neurons. n indicated in figure. Fischer's exact test P = 0.29. N = 14 mice (Ctrl), N = 8 (Cpz). All data are provided as mean ± SEM Source data are provided as a Source Data file.

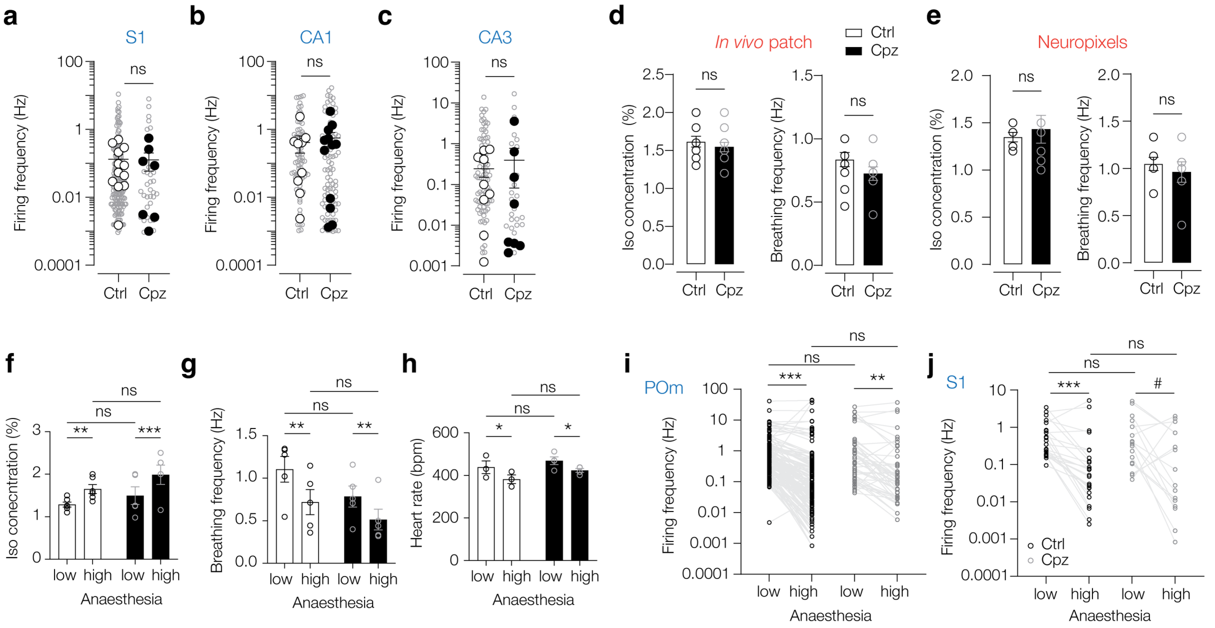

Supplementary Fig. 5 | Spontaneous firing frequencies depend on anesthesia depth but not treatment.

a Average firing frequency of single units in S1 was unchanged. Mann-Whitney test (over animal means) P = 0.71. n = 192 neurons, N = 12 mice (Ctrl), n = 34 neurons, N = 7 mice (Cpz).
b Average firing frequency of single units in CA1 was unchanged. Mann-Whitney test (over animal means) P = 0.97. n = 77 neurons, N = 9 mice (Ctrl). n = 84 neurons, N = 12 mice (Cpz).
c Average firing frequency of single units in CA3 was unchanged. Mann-Whitney test (over animal means) P = 0.42. n = 83 neurons, N = 9 mice (Ctrl), n = 26 neurons, N = 8 mice (Cpz).
d *Left*: Isoflurane concentration used to anesthetize the *in vivo* patched mice were not different between groups. Unpaired t-test P = 0.48, t = 0.724, df = 22. N = 13 (Ctrl), N = 11 (Cpz). *Right*: Breathing frequency. t-test P = 0.18, t = 1.395, df = 18. N = 11 (Ctrl), N = 9 (Cpz).
e *Left*: Isoflurane concentration used to anaesthetize the Neuropixels mice were not different between groups. Mann-Whitney test P = 0.986. N = 7 mice (Ctrl), N = 9 mice (Cpz). *Right*: Breathing frequency. t-test P = 0.52. N = 7 mice (Ctrl & Cpz).
f Effect of anesthesia was compared in additional set of recordings at low (~1.3–1.5% isoflurane) and high anesthesia (~1.7–2% isoflurane). Paired 2-way ANOVA P = 0.0001, $F_{(1,9)}$ = 40.36 anaesthesia; P = 0.2, $F_{(1,9)}$ = 1.725 treatment; P = 0.0013, $F_{(9,9)}$ = 9.439 neuron. Uncorrected Fischer's LSD **P = 0.003, ***P = 0.0008, P > 0.05. N = 6 mice (Ctrl), N = 5 (Cpz).
g Breathing rate was significantly decreased in both groups during high anesthesia recordings. Paired 2-way ANOVA P = 0.0004, $F_{(1,8)}$ = 34.77 anaesthesia, P = 0.19, $F_{(1,8)}$ = 2.01 treatment, P = 0.0013, $F_{(8,8)}$ = 11.06 neuron. Uncorrected Fischer's LSD **P < 0.01, P > 0.05. N = 5 mice (Ctrl & Cpz)
h Heart rate was significantly decreased in both groups during. Paired 2-way ANOVA P = 0.0084, $F_{(1,5)}$ = 17.74 anaesthesia; P = 0.21, $F_{(1,5)}$ = 2.12 treatment; P = 0.07, $F_{(5,5)}$ = 4.14 cell. Uncorrected Fischer's LSD P > 0.05 for all comparisons. N = 3 mice (Ctrl), N = 4 mice (Cpz).
i Firing frequency for POm neurons was significantly higher at low anesthesia levels for both Ctrl and Cpz mice. Kruskal-Wallis P < 0.0001, Dunn's multiple comparisons ***P < 0.0001 Ctrl high vs low, **P < 0.0032 Cpz high vs low, n = 160 single units, N = 4 mice (Ctrl), n = 49 single units, N = 2 mice (Cpz).
j Firing frequency for L5 neurons was higher at low anesthesia levels for Ctrl mice. A similar trend was observed for Cpz mice. Kruskal-Wallis P = 0.0002, Dunn's multiple comparisons **P = 0.006 Ctrl high vs low, $^{\#}$P = 0.08 Cpz high vs low. n = 27 single units, N = 5 mice (Ctrl), n = 17 single units, N = 3 mice (Cpz). All data are provided as mean ± SEM. Source data are provided as a Source Data file.

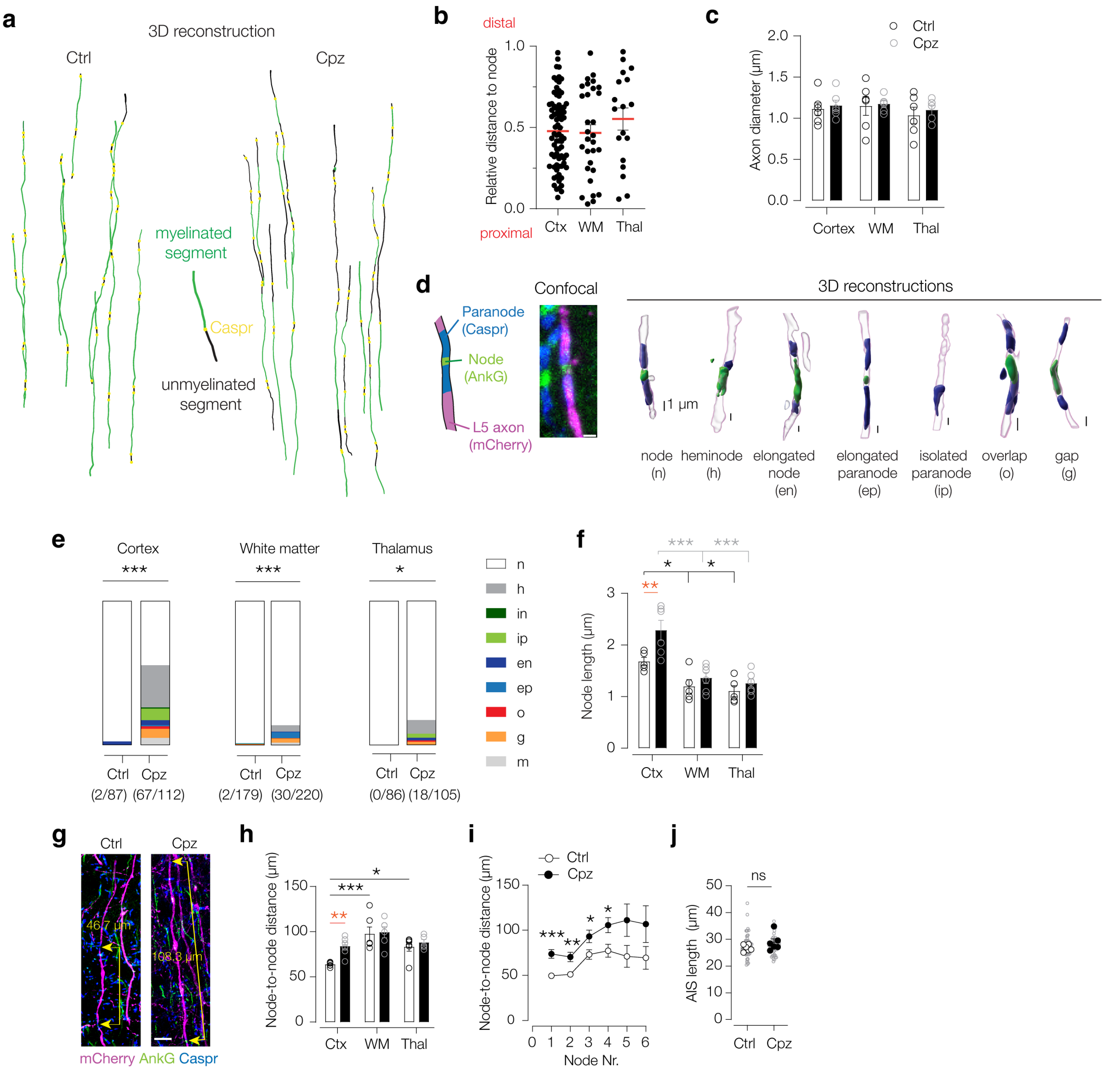

Supplementary Fig. 6 | Remodeling of axonal microdomains after demyelination.

a 3D Neurolucida reconstructions depicting the highly heterogeneous pattern of cortical demyelination of L5 axons after 6 weeks Cpz treatment.
b No preference for location of axonal spheroids within an internode (proximal or distal). One-way ANOVA P = 0.45, $F_{(2,121)}$ = 0.796. n = 76 swellings in cortex (Ctx), n = 30 white matter (WM), n = 18 thalamus (Thal), N = 8 mice.
c No change in average axon diameter after demyelination. Two-way ANOVA P = 0.52, $F_{(2,29)}$ = 0.675 treatment; P = 0.52, $F_{(1,29)}$ = 0.44 region; P = 0.97, $F_{(2,29)}$ = 0.032 interaction. Tukey's multiple comparisons test P > 0.05 for all comparisons. n = 495 axons, N = 6 mice (Ctrl), n = 445 axons, N = 6 mice (Cpz).
d 3D reconstruction of confocal images of noR depicting a variety of different aberrant morphologies after demyelination, including heminodes, isolated compartments, gaps and overlaps between node and paranode. Scale bars, 1 µm.
e Aberrant node morphologies can be found in all three regions after demyelination, most prominently in the cortex (~55%) but to a lesser extent in WM (~13%) and thalamus (~17%). c[2] test compared to control ***P < 0.0001 (Ctx), ***P< 0.0008 (WM), *P = 0.012 (Thal). Number of neurons indicated in figure. M = mixed (e.g. overlap and elongation)
f Node length was significantly increased in the cortex after Cpz. 2-way ANOVA P = 0.0064, $F_{(1,27)}$ = 8.756 treatment; P < 0.0001, $F_{(2,27)}$ = 23.63 region; P = 0.14, $F_{(2,27)}$ = 2.091 interaction. Tukey's multiple

comparisons **P < 0.0022 Ctx Ctrl vs Cpz, *P < 0.05 Ctrl Ctx vs WM & Thal, ***P < 0.0001 Cpz Ctx vs WM, Thal. n = 81, 192, 67 nodes (Ctrl Ctx, WM, Thal), n = 83, 244, 96 nodes (Cpz Ctx, WM, Thal).

e, f N = 5 mice (Ctrl), N = 6 mice (Cpz).

g Confocal image of node-to-node distance reconstructed by tracing individual axons and denoting locations of $AnkG^+$ nodes.

h Node-to-node distance was significantly increased in the cortex of Cpz mice. 2-way ANOVA P = 0.04, $F_{(1,29)}$ = 4.58 treatment; P = 0.0001, $F_{(2,29)}$ = 12.18 region; $F_{(2,29)}$ = 1.887 P = 0.17 interaction. Tukey's multiple comparisons **P = 0.0083 Ctx Ctrl vs Cpz. ***P = 0.0001 Ctrl Ctx vs WM, *P = 0.027 Ctrl Ctx vs Thal. n = 717 internodes (Ctrl), n = 558 internodes (Cpz).

i Node-to-node distances were increasingly longer with increasing distance from the soma in both Ctrl and Cpz axons. However, starting from the first internode, Cpz nodes are significantly further away. 2-way ANOVA P < 0.0001, $F_{(5,373)}$ = 11.65 node-nr.; P < 0.0001, $F_{(1,373)}$ = 31.15 treatment; P = 0.79, $F_{(5,373)}$ = 0.48 interaction. Šídák's multiple comparisons *P < 0.04, **P = 0.009, ***P = 0.0001. n = 74 internodes (Ctrl), n = 56 (Cpz).

j AIS length was unchanged between Ctrl and Cpz neurons. Nested t-test P = 0.22, t = 1.31, df = 10. n = 87 AIS (Ctrl), n = 62 AIS (Cpz).

h, i, j N = 6 mice (Ctrl & Cpz). All data are provided as mean ± SEM. Source data are provided as a Source Data file.

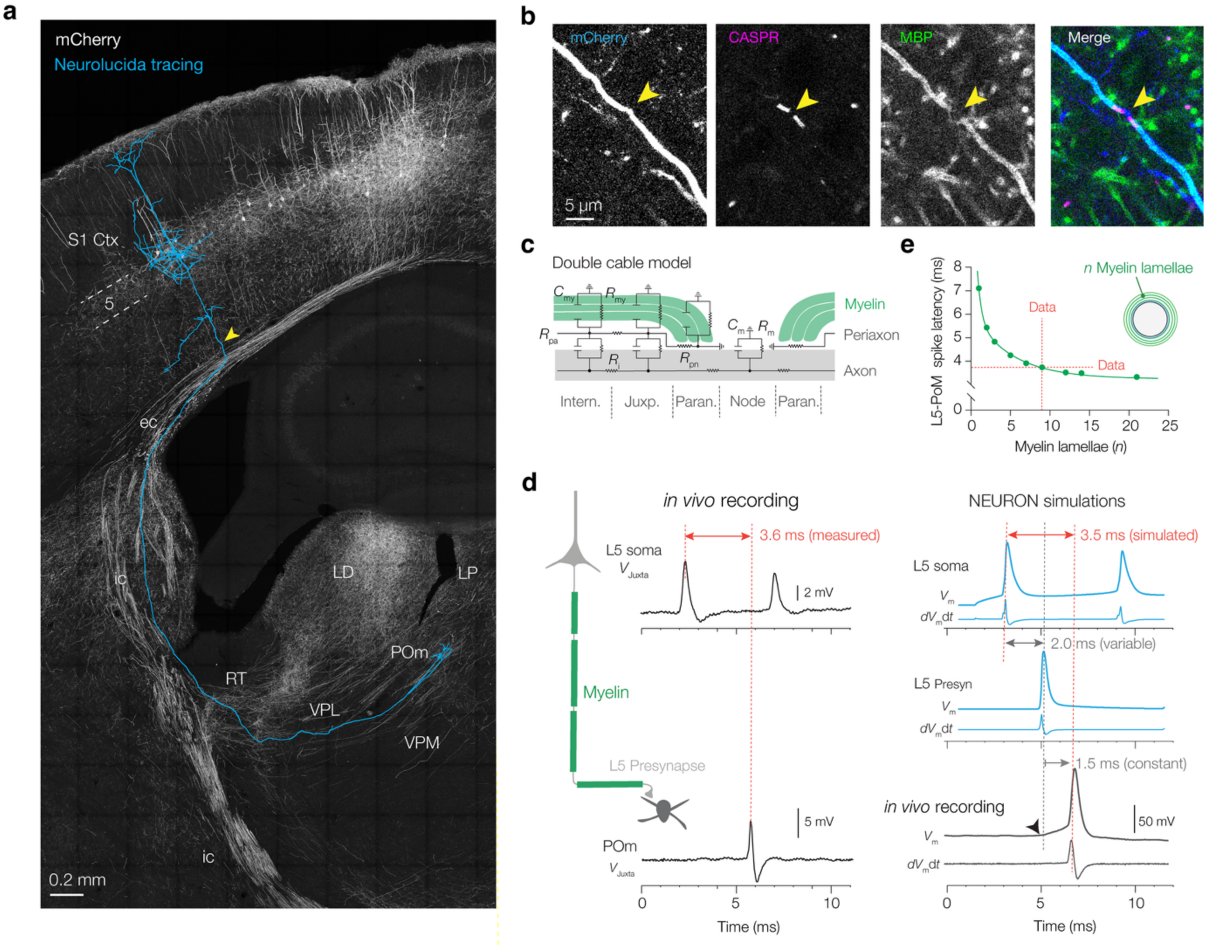

Supplementary Fig. 7 | Experimentally constrained simulations of the L5–POm delay in a myelinated axon.

a Overview of the Neurolucida tracing of the neuron (blue) used for the compartmental model, projected on a large tilescan of L5–POm mCherry+ neurons and axons. Yellow arrow indicates the nodal region shown in b.
b mCherry, MBP, AnkG and Caspr fluorescence were used to guide the assignment of biologically realistic dimensions of myelinated sections and nodal domains. The model neuron comprised of 515 sections including 63 internodes and 49 nodes of Ranvier.
c Double-cable circuit diagram of the nodal domain showing the electrical resistor-capacitor pathways to represent the node, paranode (paran.), juxtaparanode (juxp.) and internode (intern.) compartment.
d *Left*, cartoon of the L5–POm neuron with example optotagged juxtacellular recordings from L5 or POm soma, aligned to the optogenetic stimulus onset. Spike latency for this recording was 3.6 ms. *Right*, simulations of voltage-time (*V*-*t*) and temporal derivative ($dV \cdot dt^{-1}$) of APs evoked by a 20-ms current injection (1.7 nA) showing the first two APs of a ~200 Hz burst cluster. Soma to presynaptic spike delay was 2.0 ms. The L5–POm spike-spike latencies were calculated by adding a nominal value of 1.5 ms. A constant of 1.5 ms was predicted by POm *in vivo* whole-cell recordings, revealing onset of EPSP to the peak $dV \cdot dt^{-1}$ of the spike being on average 1.5 ms (lower trace), and also in accord with photo-evoked L5–POm EPSPs and spikes being temporally separated by 1.5 ms [27].
e L5–POm latencies varied with simulated myelin parameter (C_{my} and R_{my}). Red dotted lines indicate a delay time of 3.5 ms with 9 myelin lamellae, consistent with anatomical data [31,42,43]. Source data are provided as a Source Data file.

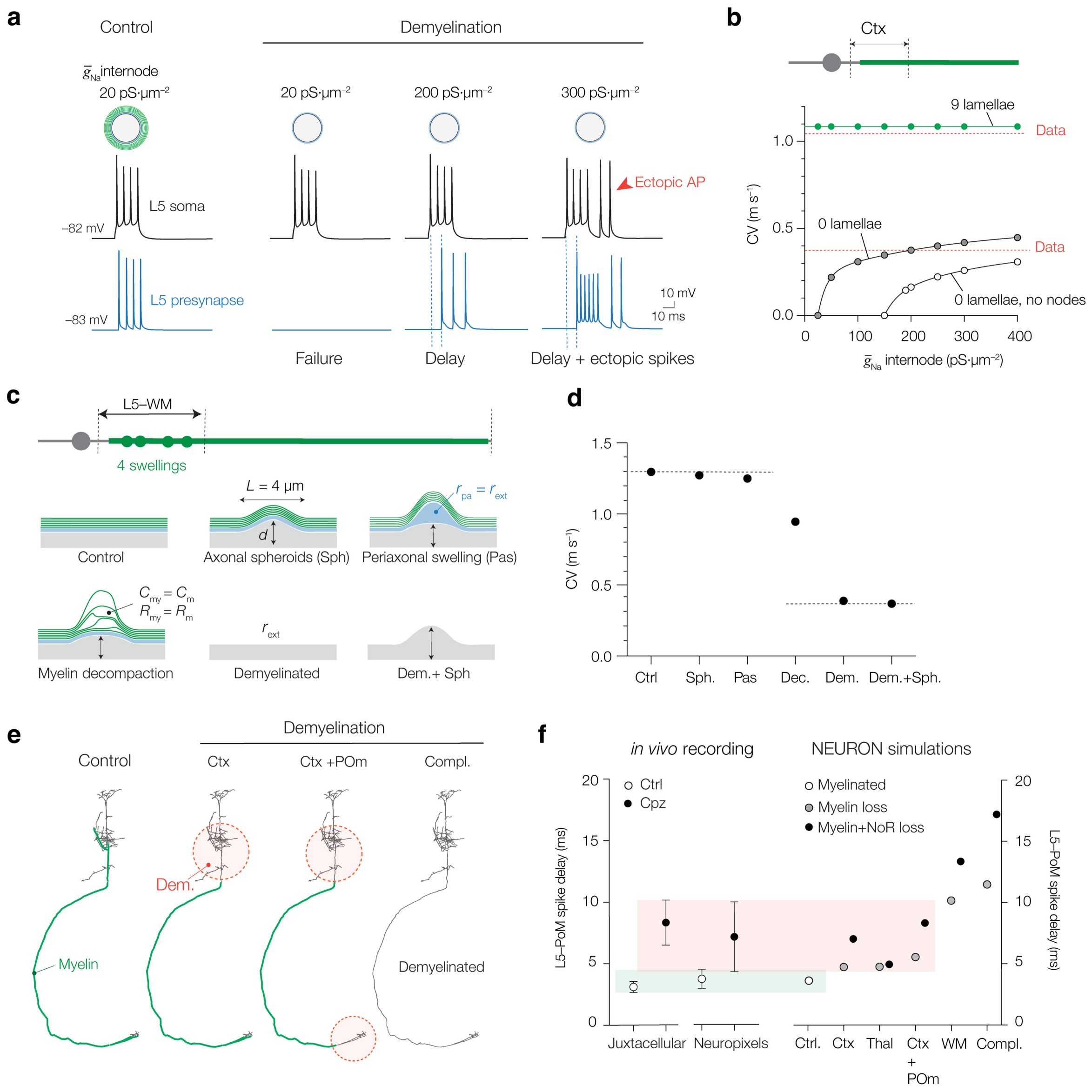

Supplementary Fig. 8 | Simulating demyelination of the L5–POm axon.

a *Left*, Example model traces of AP burst evoked by current injection in the soma (black, 1.7 nA, not shown) propagating into the giant synapse (blue) in control conditions [9 myelin lamellae, green and sodium peak conductance density ($\bar{g}_{Na}$) internode = 20 pS·µm^{-1}]. *Right*, removing myelin but leaving nodal compartments intact causes a complete spike failure, rescued by increasing in $\bar{g}_{Na}$ in the internodal compartments. If $\bar{g}_{Na}$> 200 pS·µm^{-1} ectopic spikes (red arrow) were initiated which propagated antidromic and orthodromic.
b CV versus internodal $\bar{g}_{Na}$ in control (green), demyelinated axons with intact nodes (0 lamellae, grey circles) and demyelinated axons without nodes (open circles). In control ~0.5 mm distance from the soma the CV along the myelinated primary axon was 1.09 m·s^{-1}, well in accord with previous patch-clamp recordings from L5 axons (1.1 m·s^{-1}, Data from Ref. [19]. Following cuprizone treatment neocortical CV reduces to 0.35 m·s^{-1}, Data from [19,20]. A $\bar{g}_{Na}$ value of 200 pS·µm^{-1} was selected for models of demyelination, below the threshold of ectopic AP generation and consistent with experimentally measured CVs in demyelinated axons [19,20].
c Axonal spheroids were introduced at three internode sites in the cortex and one in WM. Model parameters included locally and linearly increased and decreased axon diameter (*d*) from 1.5 to 7 µm, over a length (*L*) of 4 µm. Additional periaxonal swelling was simulated by lowering local r_{pa} to extracellular resistivity values of 1×10^{-6} MΩ·cm^{-1}. Local myelin decompaction of the spheroids was simulated by setting C_{my} and R_{my} locally to neuronal membrane values.

d CV determined from AIS to a node of Ranvier in the WM (700 μm from the soma). Note that the local myelin decompaction along only ~16 μm length (only 2.5% of the myelinated path) substantially impeded velocity.
e Illustrations of the regional demyelination (red areas) implemented in the model, reproducing myelin loss in Ctx and POm, as observed in **Fig. 5**, **Supplementary Fig. 6.**
f Bar plot of L5–POm time delays for various demyelinated regions of the axon (grey circles) or demyelination with node loss (black circles). Control delay (white circle) is within range of experimentally determined velocities (mean and S.D.) and the delays measured in cuprizone overlaps with a demyelinated Ctx and POm region (red shaded area), but not WM or a completely demyelinated axon. Source data are provided as a Source Data file.

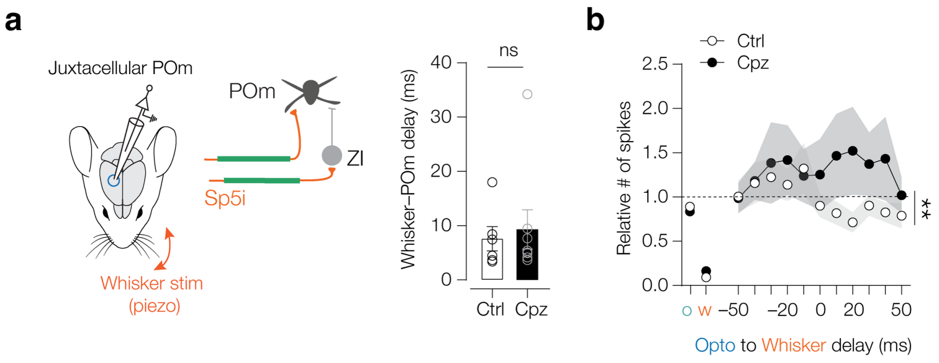

Supplementary Fig. 9 | Demyelination of corticothalamic feedback alters the temporal encoding of whisker stimulation.

a Invariant whisker-evoked delay in "early" responder neurons after demyelination. Mann-Whitney test P = 0.75. n = 6 neurons, N = 5 mice (Ctrl), n = 8 neurons, N = 7 mice (Cpz). (mouse brain adapted from [103]).

b Population average shows a preferential increase in spike numbers if the opto stim precedes the whisker stim for Ctrl but not for Cpz neurons. 2-way ANOVA P = 0.0082, $F_{(1,318)}$ = 7.082 treatment; P = 0.0007, $F_{(12,318)}$ = 2.918 delay; P = 0.79, $F_{(12,318)}$ = 0.654 interaction. Šídák's multiple comparisons test P > 0.05 for all comparisons. n = 15 neurons, N = 11 mice (Ctrl), n = 13 neurons, N = 7 mice (Cpz). All data are provided as mean ± SEM. Source data are provided as a Source Data file.

Supplementary Table 1. Antibody details

Antibody	Host	Dilution	Manufacturer	Cat. #.	RRID
Anti-Myelin Basic Protein	mouse	1:250	Covance	SMI-99P-100	AB_10120129
Anti-Red fluorescent protein	Chicken	1:1000	Synaptic Systems	409 006	AB_2725776
Anti-Red fluorescent protein	Guinea pig	1:1000	Synaptic Systems	390004	AB_2737052
Anti-Caspr	Rabbit	1:1000	Abcam	ab34151	AB_869934
Anti-Ankyrin G	Guinea pig	1:500	Synaptic Systems	386 004	AB_2725774
Streptavidin-Alexa488	/	1:500	Thermo Fischer	S11223	/
Streptavidin-Alexa594	/	1:500	Thermo Fischer	S11227	/
Anti-Rabbit Alexa405	Goat	1:1000	Thermo Fischer	A31556	AB_221605
Anti-mouse Alexa488	Goat	1:1000	Thermo Fischer	A10684	AB_2534064
Anti-guineapig Alexa488	Goat	1:1000	Thermo Fischer	A11073	AB_2534117
Anti-chicken Alexa594	Goat	1:1000	Thermo Fischer	A11042	AB_2534099
Anti-guineapig Alexa633	Goat	1:500	Thermo Fischer	A21105	AB_2535757
Anti-guineapig Alexa647	Goat	1:1000	Thermo Fischer	A21450	AB_2535867
