# Supplementary material for: Layer 5 myelination gates corticothalamic coincidence detection
Source: Nat Commun. 2025 Dec 11;16:10922. doi: 10.1038/s41467-025-66157-1 (PMC12699038; doi:10.1038/s41467-025-66157-1)
Supplement: Supplementary file 2 — Description of Additional Supplementary Files [file 41467_2025_66157_MOESM2_ESM.pdf]

Description of Additional Supplementary Files

File Name: Supplementary Movie 1
Description: 3D visualization of a stitched and fused light-sheet image showing the sparsely labelled L5 population (*magenta)* projecting to the spinal cord. One single axon was manually reconstructed from L5 continuing into the proximal spinal cord as well as an axon branch projecting to the POm (*white*), overlaid it with the imaged brain. Scale bar, 2 mm.

File Name: Supplementary Movie 2
Description: Spatial profile of transaxonal (internodes) and transmembrane potentials (somatodendritic regions and noR) temporally aligned with the voltage-time plots of the soma (*black*) and giant terminal in the POm (*red*). A schematic version of the neuronal morphology and myelin (*green*) is shown at the top. For clarity, only transaxonal is shown, but not the transmyelin and transfiber potentials. Movie shows a high-frequency burst (evoked by a 20-ms current injection) propagating along the control myelinated axon in rapid saltatory mode (1.93 $m \cdot s^{-1}$). Subsequent movie shows the example of cortical demyelination (slow continuous conduction, 0.35 $m \cdot s^{-1}$). Red arrows indicate demyelinated site and the propagation failure in the voltage-time plots of the terminal. Subsequent movie shows an expanded spatial scale revealing spike propagation block at the first noR. Final movie, axonal Na_v conductance density increased from 200 to 300 $pS \cdot \mu m^{-2}$, revealing ectopic APs starting from the second noR. Temporal resolution of simulations is 0.01 ms.

File Name: Supplementary Data 1
Description: Complete overview of the statistical test results and parameter values per figure.
