# Supplementary material for: Layer 5 myelination gates corticothalamic coincidence detection
Source: Nat Commun. 2025 Dec 11;16:10922. doi: 10.1038/s41467-025-66157-1 (PMC12699038; doi:10.1038/s41467-025-66157-1)
Supplement: Supplementary file 6 — Reporting Summary [file 41467_2025_66157_MOESM6_ESM.pdf]

nature portfolio

Corresponding author(s): Maarten H.P. Kole

Last updated by author(s): Oct 25, 2025

Reporting Summary

Nature Portfolio wishes to improve the reproducibility of the work that we publish. This form provides structure for consistency and transparency in reporting. For further information on Nature Portfolio policies, see our Editorial Policies and the Editorial Policy Checklist.

Statistics

For all statistical analyses, confirm that the following items are present in the figure legend, table legend, main text, or Methods section.

n/a	Confirmed	
☐	☒	The exact sample size (*n*) for each experimental group/condition, given as a discrete number and unit of measurement
☐	☒	A statement on whether measurements were taken from distinct samples or whether the same sample was measured repeatedly
☐	☒	The statistical test(s) used AND whether they are one- or two-sided<br>*Only common tests should be described solely by name; describe more complex techniques in the Methods section.*
☐	☒	A description of all covariates tested
☐	☒	A description of any assumptions or corrections, such as tests of normality and adjustment for multiple comparisons
☐	☒	A full description of the statistical parameters including central tendency (e.g. means) or other basic estimates (e.g. regression coefficient) AND variation (e.g. standard deviation) or associated estimates of uncertainty (e.g. confidence intervals)
☐	☒	For null hypothesis testing, the test statistic (e.g. *F*, *t*, *r*) with confidence intervals, effect sizes, degrees of freedom and *P* value noted<br>*Give P values as exact values whenever suitable.*
☒	☐	For Bayesian analysis, information on the choice of priors and Markov chain Monte Carlo settings
☒	☐	For hierarchical and complex designs, identification of the appropriate level for tests and full reporting of outcomes
☒	☐	Estimates of effect sizes (e.g. Cohen's *d*, Pearson's *r*), indicating how they were calculated

Our web collection on statistics for biologists contains articles on many of the points above.

Software and code

Policy information about availability of computer code

Data collection	Leica Application Suite AF (version 3.5.7.23225)<br>STEDYCON software (Version 9, Abberior Instruments, Göttingen, Germany)<br>Imspector software (version V380, LaVision BioTec)<br>PClamp software (Version 10.7, Molecular Devices)<br>Axograph X (v.1.5.4, Axograph, RRID:SCR_014284, NSW, Australia)<br>SpikeGLX (https://github.com/billkarsh/SpikeGLX, Version 20201103)
Data analysis	Neurolucida (version 2020.1.3, MBF Bioscience, RRID: SCR_001775)<br>Imaris (Version 9.6.1, Oxford Instruments, Abingdon, England, RRID SCR_007370)<br>ImageJ (FIJI; ImageJ version 1.54f; RRID: SCR_003070)<br>https://github.com/Kolelab/Neuropixels_ephys_Jamann_2025 (Custom code repository)<br>BigStitcher Plugin in ImageJ (Version 2.5.2.), https://imagej.net/plugins/bigstitcher<br>simple neurite tracer (Version 4.2.1, https://imagej.net/plugins/snt/)<br>sci-kit image (Version 0.22.2, https://scikit-image.org/)<br>Napari (https://napari.org/stable/, https://zenodo.org/doi/10.5281/zenodo.3555620)<br>Axograph X (v.1.5.4, Axograph, RRID:SCR_014284, NSW, Australia)<br>Kilosort (v2.5 https://github.com/MouseLand/Kilosort)<br>bombcell (V1.5.0, https://github.com/Julie-Fabre/bombcell)<br>Phy (V2.0a1, https://github.com/cortex-lab/phy)

April 2023

Universal Probe Finder (V1.1.1, https://github.com/JorritMontijn/UniversalProbeFinder)
Acquipix (V0.9.0, https://github.com/JorritMontijn/Acquipix)
AP histology (V2, https://github.com/petersaj/AP_histology)
ZETA (https://github.com/JorritMontijn/ZETA)
NEURON simulation environment (v.7.8.2, RRID:SCR_005393)
GraphPad Prism 8 software (GraphPad Software, Inc., Version 10.2.3., RRID SCR_002798)
Matlab (depending on the toolbox or analysis 2018a, 2021b or 2023b, MathWorks, Massachusetts, USA, RRID SCR_001622)

For manuscripts utilizing custom algorithms or software that are central to the research but not yet described in published literature, software must be made available to editors and reviewers. We strongly encourage code deposition in a community repository (e.g. GitHub). See the Nature Portfolio guidelines for submitting code & software for further information.

Data

Policy information about availability of data

All manuscripts must include a data availability statement. This statement should provide the following information, where applicable:

- Accession codes, unique identifiers, or web links for publicly available datasets
- A description of any restrictions on data availability
- For clinical datasets or third party data, please ensure that the statement adheres to our policy

The datasets generated during and/or analysed during the current study are available from the corresponding author.

Research involving human participants, their data, or biological material

Policy information about studies with human participants or human data. See also policy information about sex, gender (identity/presentation), and sexual orientation and race, ethnicity and racism.

Reporting on sex and gender	N/A
Reporting on race, ethnicity, or other socially relevant groupings	N/A
Population characteristics	N/A
Recruitment	N/A
Ethics oversight	N/A

Note that full information on the approval of the study protocol must also be provided in the manuscript.

Field-specific reporting

Please select the one below that is the best fit for your research. If you are not sure, read the appropriate sections before making your selection.

☒ Life sciences ☐ Behavioural & social sciences ☐ Ecological, evolutionary & environmental sciences

For a reference copy of the document with all sections, see nature.com/documents/nr-reporting-summary-flat.pdf

Life sciences study design

All studies must disclose on these points even when the disclosure is negative.

Sample size	We used a power analysis, where a certain sample size generated a dataset for analysis with sufficent power ((P > 0.8) based on an expected effect size and variance of the measured parameter.
Data exclusions	No data were excluded
Replication	Findings were replicated in sets of 2-3 mice that were treated and analyzed sequentially.
Randomization	Mice from the same litters were randomly assigned to the control or treatment group.
Blinding	Investigators were not blinded during data collection or analysis. However, the data was processed with the same code and thus in an unbiased way. For morphology, blinding was not possible since the treatment effect (demyelination) can be readily observed during the performance of the analysis.

Reporting for specific materials, systems and methods

We require information from authors about some types of materials, experimental systems and methods used in many studies. Here, indicate whether each material, system or method listed is relevant to your study. If you are not sure if a list item applies to your research, read the appropriate section before selecting a response.

Materials & experimental systems

n/a	Involved in the study
	[x] Antibodies
[x]	Eukaryotic cell lines
[x]	Palaeontology and archaeology
	[x] Animals and other organisms
[x]	Clinical data
[x]	Dual use research of concern
[x]	Plants

Methods

n/a	Involved in the study
[x]	ChIP-seq
[x]	Flow cytometry
[x]	MRI-based neuroimaging

Antibodies

Antibodies used

Name, Host species, Dilution, Source, Ct-Nr., RRID
Anti-Myelin Basic Protein, Mouse, 1:250, Covance, SMI-99P, AB_10120129
Anti-Red fluorescent protein, Chicken, 1:1000, Synaptic Systems, 409 006, AB_2725776
Anti-Red fluorescent protein, Guinea pig, 1:1000, Synaptic Systems, 390004, AB_2737052
Anti-Caspr, Rabbit ,1:1000, Abcam, ab34151, AB_869934
Anti-Ankyrin G, Guinea pig, 1:500, Synaptic Systems, 386 004, AB_2725774
Streptavidin-Alexa488, 1:500, Thermo Fischer, S11223
Streptavidin-Alexa594, 1:500, Thermo Fischer, S11227
Anti-Rabbit Alexa405, Goat, 1:1000, Thermo Fischer, A31556, AB_221605
Anti-mouse Alexa488, Goat, 1:1000, Thermo Fischer, A10684, AB_2534064
Anti-guineapig Alexa488, Goat, 1:1000, Thermo Fischer, A11073, AB_2534117
Anti-chicken Alexa594, Goat, 1:1000, Thermo Fischer, A11042, AB_2534099
Anti-guineapig Alexa633, Goat, 1:500, Thermo Fischer, A21105, AB_2535757
Anti-guineapig Alexa647, Goat, 1:1000, Thermo Fischer, A21450, AB_2535867

Validation

Validation as indicated by manufacturer websites:
Anti-Myelin Basic Protein: validated by Covance via immunofluorescence (IF) and western blot (WB)
Anti-Red fluorescent protein: validated by Synaptic Systems via IF
Anti-Red fluorescent protein: validated by Synaptic Systems via IF and WB
Anti-Caspr: validated by Abcam via IF, WB and immunoprecipitation, validated in a KO.
Anti-Ankyrin G: validated by Synaptic Systems via IF and WB

Animals and other research organisms

Policy information about studies involving animals; ARRIVE guidelines recommended for reporting animal research, and Sex and Gender in Research

Laboratory animals

The strain used for this study was B6.FVB(Cg)-Tg(Rbp4-cre)KL100Gsat/Mmucd, RRID:MMRRC_037128-UCD (Rbp4-Cre).
Age range of animals was between 12 and 17 weeks at sacrifice.

Wild animals

N/A

Reporting on sex

Both male and female mice were used for this study and randomly assigned to treatment and control groups

Field-collected samples

N/A

Ethics oversight

All procedures were performed after evaluation by the KNAW Animal Ethics Committee (DEC) and Central Authority for Scientific Procedures on Animals (CCD, license AVD-80100202216329). The specific experimental designs were evaluated and monitored by the Animal Welfare Body (IvD, protocols NIN 20.21.04, NIN 21.21.04).

Note that full information on the approval of the study protocol must also be provided in the manuscript.

Plants

Seed stocks	N/A
Novel plant genotypes	N/A
Authentication	N/A
